# Linking Electrical Drivers With Atrial Cardiomyopathy for the Targeted Treatment of Atrial Fibrillation

**DOI:** 10.3389/fphys.2020.570740

**Published:** 2020-11-12

**Authors:** Gordon Ho, Andrew Y. Lin, David E. Krummen

**Affiliations:** ^1^Division of Cardiology, Department of Medicine, University of California, San Diego, San Diego, CA, United States; ^2^Division of Cardiology, Veterans Affairs San Diego Medical Center, San Diego, CA, United States

**Keywords:** atrial fibillation, fibrosis, cardiac imaging, electrophysiologic mapping, electrical rotors, focal sources

## Abstract

The relationship between atrial fibrillation (AF) and underlying functional and structural abnormalities has received substantial attention in the research literature over the past decade. Significant progress has been made in identifying these changes using non-invasive imaging, voltage mapping, and electrical recordings. Advances in computed tomography and cardiac magnetic resonance imaging can now provide insight regarding the presence and extent of cardiac fibrosis. Additionally, multiple technologies able to identify electrical targets during AF have emerged. However, an organized strategy to employ these resources in the targeted treatment of AF remains elusive. In this work, we will discuss the basis for mechanistic importance of atrial fibrosis and scar as potential sites promoting AF and emerging technologies to identify and target these structural and functional substrates in the electrophysiology laboratory. We also propose an approach to the use of such technologies to serve as a basis for ongoing work in the field.

## Introduction

Atrial fibrillation (AF) is the most common cardiac arrhythmia in the United States (Chugh et al., 2014). Catheter ablation is offered for patients with symptomatic AF despite medical therapy (January et al., 2014, 2019; Calkins et al., 2017), but success rates for ablation of persistent AF continues to be suboptimal with recurrent AF occurring in around 40–60% of patients in the landmark CABANA (Packer et al., 2019) and STAR-AF2 (Verma et al., 2015) trials. Potential contributing factors to the suboptimal success rates are the diverse phenotypes of atrial structural and functional abnormalities seen in patients with AF (Kottkamp, 2013; Krummen et al., 2015). While emerging technologies are now able to detect, classify, and target abnormal atrial substrate, their use is not well guided by existing guidelines or supported by randomized clinical trials (Quintanilla et al., 2016; Calkins et al., 2017). The purpose of this review is to propose a personalized AF ablation strategy utilizing emerging mapping and imaging techniques to target electrical drivers with or without associated atrial fibrosis. First, electrical and structural mechanisms of AF are summarized, followed by a review of the evidence linking fibrosis with electrical drivers of AF. Second, contemporary electrical invasive and non-invasive mapping and imaging techniques are discussed to localize electrical drivers and fibrosis. Finally, a proposed algorithm is proposed to help guide personalized clinical treatment using these technologies and guide further clinical research.

## Diverse Atrial Substrates Underlying AF

**FIGURE 1:**
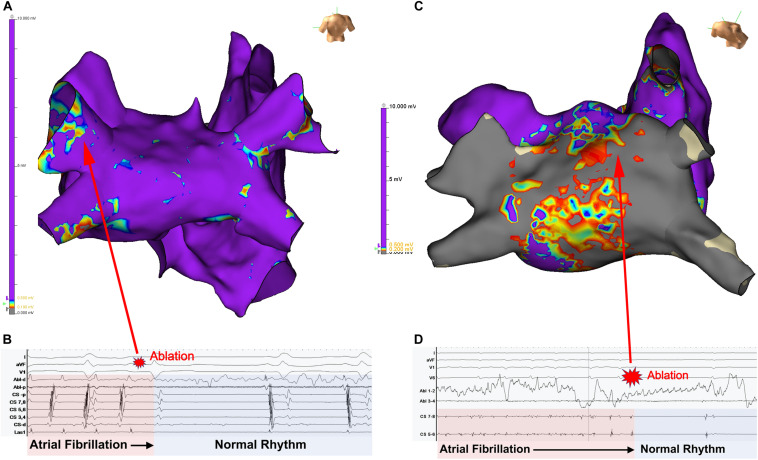
Panel **(A)** shows the left atrial voltage map of a patient with persistent atrial fibrillation for 4 months. Panel **(B)** illustrates electrogram recordings from the ablation catheter showing a rapid driver site in the left upper pulmonary vein. Ablation at this site abruptly terminated AF. Panel **(C)** Bi-atrial voltage map of a patient with persistent atrial fibrillation s/p prior ablation with diffuse low-voltage and scar in the left atrium. Panel **(B)** shows a left atrial driver site in the LA roof. Panel **(D)** shows termination of AF with targeted ablation. Unpublished work from our laboratory.

In clinical practice, AF patients present with a spectrum of atrial electrical and structural substrates (Everett and Olgin, 2007; Kottkamp, 2013; Goette et al., 2016). Figure 1 compares two patients from our electrophysiology laboratory with contrasting degrees of atrial cardiomyopathy. Patient 1 presented with persistent AF who remained symptomatic despite medical management and was referred for ablation. Voltage and activation mapping revealed relatively preserved bi-atrial voltages (Figure 1A) and a rapid left upper pulmonary vein electrical driver perpetuating AF (Figure 1B). Localized ablation terminated AF, which was subsequently non-inducible. In this patient with normal structural substrate, the AF was likely driven by an electrical driver from the pulmonary veins, as classically described (Haïssaguerre et al., 1998).

**FIGURE 2:**
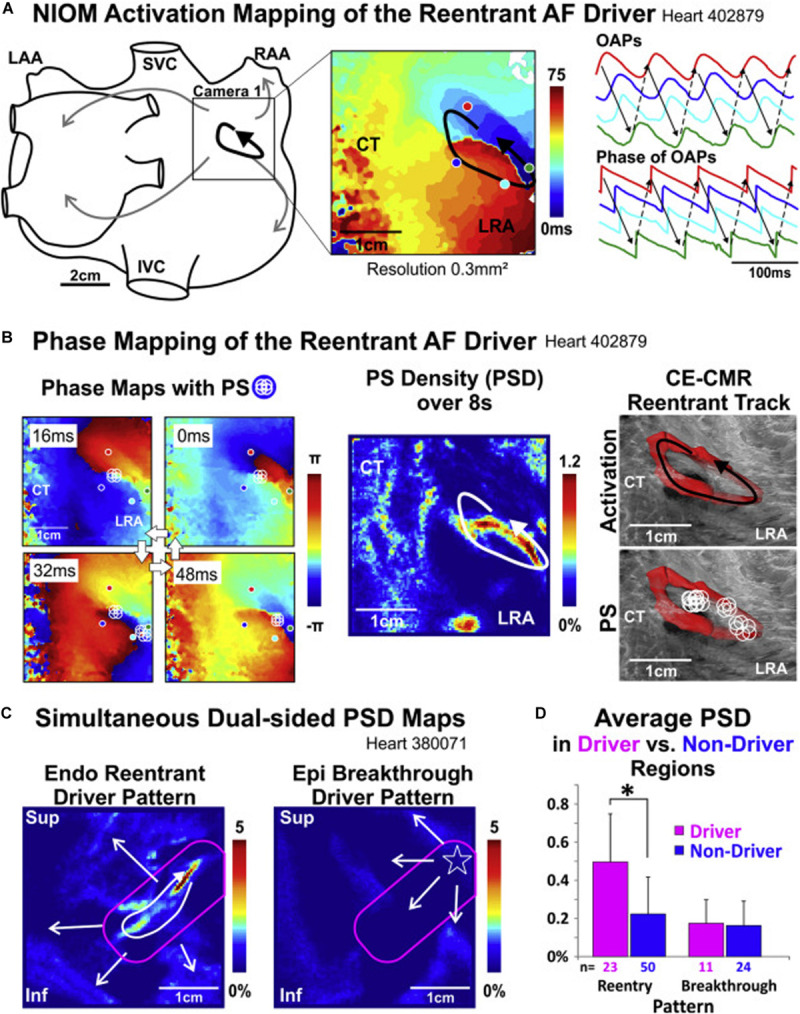
Re-entrant drivers visualized with high resolution optical activation mapping **(A)** are colocalized with endocardial phase mapping using FIRMap basket catheters [**(B)**, left and center panels], and are anchored to regions of intramural fibrosis imaged with 9.4 T MRI [**(B)**, right panel]. Notably, endocardial re-entrant drivers result in epicardial focal breakthrough patterns when imaged with simultaneous endo-epi phase mapping **(C)**, with higher phase singularity density in regions with reentrant drivers identified **(D)**. NIOM, near-infrared optical mapping; CT, crista terminalis; LRA, low right atrium; OAP, optical action potentials; PS, phase singularities; CE-CMR, contrast-enhanced magnetic resonance; Endo, endocardium; Epi, epicardium; Inf, inferior; IVC, inferior vena cava; LAA, left atrial appendage; LRA, lateral right atrium; RAA, right atrial appendage; Sup, superior; SVC, superior vena cava. Hansen et al. (2018), reprinted with permission.

Patient 2 presented with recurrent persistent AF despite prior pulmonary vein isolation ablation. Voltage mapping revealed diffuse low voltage in the left atrium (Figure 1C), while panoramic multielectrode catheter mapping identified a rotational AF driver at the LA roof (Figure 1D). Limited ablation in the region of this driver terminated AF (Figure 2C), and the patient has remained in sinus rhythm during follow-up. In this patient with significant atrial fibrosis, extra-pulmonary vein drivers arising from the fibrotic substrate likely contributed to AF maintenance. These two examples demonstrate a broad spectrum of the structural and electrical substrate that may underly AF. While increasing atrial fibrosis typically correlates with a greater prevalence of extra-pulmonary vein sources (Angel et al., 2015; Cochet et al., 2018), counterintuitively, patients without fibrosis may have persistent AF while patients with extensive atrial fibrosis due to atrial cardiomyopathy may only have brief paroxysms of AF (Kottkamp, 2013). In a study by Kircher et al. (2018) in which invasive substrate mapping was performed in 119 patients, only 40% of persistent AF patients had low voltage zones, yet low voltage zones were still found in 18% of paroxysmal AF patients. While the AF source in the first patient would have been accounted for with guideline-directed pulmonary vein isolation (Haïssaguerre et al., 1998; Narayan et al., 2008), the driver located at the left atrial roof in patient 2 would not. Such examples, prevalent in the literature (Narayan et al., 2012a; Shivkumar et al., 2012) demonstrate the need for additional guidance regarding the use of patient-specific mapping and targeting strategies to treat AF.

## Mechanisms of Atrial Fibrillation

There remains controversy surrounding the exact mechanisms of AF. This is likely due to multiple phenotypes of the arrhythmia and reflects a heterogeneous substrate (Krummen et al., 2015; Quintanilla et al., 2016; Calkins et al., 2017). Both abnormal electrical (Krummen et al., 2012; Baykaner et al., 2014, 2018; Haissaguerre et al., 2014; Schricker et al., 2014; Quintanilla et al., 2016) and structural/fibrotic abnormalities (Everett and Olgin, 2007; McDowell et al., 2013, 2015; Gonzales et al., 2014; Hansen et al., 2015, 2018; Sohns and Marrouche, 2020) have been implicated as mechanisms of AF, and may be different for each patient.

### Electrical Substrate

Abnormal electrical substrate underlying AF may exist with or without the presence of fibrosis, and can be divided into 3 phases: initiation, transition and maintenance (Heijman et al., 2014; Krummen et al., 2015).

1. Initiation: The initiation of AF is thought to arise from rapid activation from a source, including the pulmonary veins (Haïssaguerre et al., 1998), superior vena cava, or other rapid foci (Gerstenfeld et al., 2003; Lin et al., 2003; Chen and Tai, 2005; Van Campenhout et al., 2013; Hayashi et al., 2015). This rapid ectopic activity can be caused by triggered activity [abnormalities in calcium handling leading to delayed afterdepolarizations (Dobrev et al., 2011) or loss of K + currents and delayed repolarization leading to early afterdepolarizations (Zellerhoff et al., 2009)] and abnormal automaticity, which have all been described around the LA/PV junction (Chou et al., 2005; Numata et al., 2012). At present, the standard of care for AF ablation is directed primarily at targeting triggers of AF from the pulmonary veins only. While reasonably effective, long-term elimination of AF triggers may be difficult or impossible, depending upon the rate of trigger formation, the dispersal of AF sources, and their frequency during invasive mapping and ablation procedures.
2. Transition: The transition period is the time during which the rapid activations from a focal source interact with regions with heterogeneous repolarization properties resulting in regional wavefront block, wavefront slowing, and the initiation of reentry (Krummen et al., 2012; Lalani et al., 2012; Schricker et al., 2014). This tissue heterogeneity can be manifested by both spatial dispersion of atrial fibrosis (Li et al., 1999; Chang et al., 2007) and ion currents [such as density of the rapid delayed rectifier current IKr (Li et al., 2001)], causing differences in conduction slowing, tissue refractoriness, and steep APD restitution (Krummen et al., 2012) to favor reentry.
3. Maintenance: Although the precise mechanisms of AF maintenance are incompletely understood, there is increasing evidence that AF is maintained by organized mechanisms. The exact type of organization is a topic of controversy, as some groups have proposed that AF is maintained via dissociated endo-epicardial breakthroughs (de Groot et al., 2010) or focal or rotational drivers (Jalife, 2003; Baykaner et al., 2014; Haissaguerre et al., 2014; Lalani et al., 2014; Schricker et al., 2014; Hansen et al., 2015, 2017, 2018; Quintanilla et al., 2016; Zahid et al., 2016; Csepe et al., 2017; Nattel et al., 2017; Zhao et al., 2017). Recent seminal work has demonstrated that rotational and focal drivers exist at sites that terminate AF when ablated, reinforcing the role of organized drivers maintain (Zhao et al., 2017; Kowalewski et al., 2018; Zaman et al., 2018; Leef et al., 2019).

### Contribution of Fibrosis to Tissue Electrical Remodeling

Fibrotic atrial myopathy is associated with alterations in ionic currents, calcium cycling, and gap junctions leading to electrophysiologic remodeling and increased atrial susceptibility to triggered activity, automaticity, and reentry (Nattel et al., 2008; Shen et al., 2019). First, triggered activity may result from direct myofibroblast-cardiomyocyte interactions via gap junction coupling and diastolic depolarization of atrial myocytes by fibroblasts (Yue et al., 2011; Heijman et al., 2014). Secondly, fibroblast ion channel remodeling may also promote AF, with increased expression of Ca + + permeable TRPC3 channels and direct myofibroblast-cardiomyocyte interactions which cause conduction slowing due to Na + channel inactivation and impaired cell-cell coupling (Heijman et al., 2014). Changes in ionic channel properties occur with significant heterogeneity between the left and right atria, which may explain the propensity of AF to originate from the left atrium (Caballero et al., 2010). Remodeling of gap junctions such as connexin 40 and 43 and their expression, distribution, and intercellular orientation in atrial myopathy causing anisotropic conduction leading to reentry has been attributed to sustained AF (Shin et al., 2015). Finally, fibrosis and collagen deposition directly causes conduction slowing and heterogeneity.

### Linking Fibrosis to Electrical AF Drivers

Advances in understanding the effects of fibrosis on electrical remodeling described above has provided a cellular basis that support recent observations correlating regions of fibrosis with focal and reentrant AF drivers. Prior work has demonstrated the relationship between functional electrical reentry and atrial structural heterogeneities such as fibrosis (Morgan et al., 2016) and fiber-angle discontinuities (Gonzales et al., 2014). Elegant *ex vivo* studies by Fedorov and colleagues reveal that AF re-entrant drivers are anchored to micro-anatomic regions of interstitial fibrosis (Hansen et al., 2015, 2016, 2017, 2018; Csepe et al., 2017; Zhao et al., 2017). In explanted human bi-atrial tissue sections shown in Figure 2, reentrant drivers were identified with high resolution optical activation mapping (Figure 2A) and colocalized using endocardial basket catheters (Figure 2B, left and center panels) with a clinical phase mapping system (FIRM, Abbott, Illinois) (Hansen et al., 2018). These drivers were anchored in regions of interstitial fibrosis imaged using high resolution 9T MRI (Figure 2B, right panel). Notably, epicardial electrodes revealed epicardial breakthroughs at sites of the endocardial reentrant drivers (Figure 2C). Ablation of these re-entrant drivers terminated AF, verifying their dominant role in AF (Hansen et al., 2015; Zhao et al., 2017). These findings confirm that the rotational drivers identified with phase mapping truly exist with high resolution optical activation mapping and reconcile how intramural rotational drivers may result in epicardial breakthroughs. Furthermore, these findings link these electrical drivers with regions of fibrosis identified with high resolution MRI.

Likewise, clinical studies using non-invasive electrocardiographic imaging (ECGi) (Cochet et al., 2018) also correlated re-entrant drivers to late gadolinium-enhanced (LGE) areas on MRI. However, other clinical studies failed to correlate rotational drivers identified using invasive phase mapping with LGE on MRI (Chrispin et al., 2016) and electroanatomic voltage mapping (Schade et al., 2016). This discrepancy may reflect differences in mapping and imaging technologies, but may also highlight the possibility that electrical drivers can arise from (1) a purely electrical substrate [electrical remodeling altering cellular gap junction distribution (Fry et al., 2014; Shin et al., 2015) without fibrosis or shortened atrial refractory period (Wijffels et al., 1995)] or (2) structural heterogeneities such as fiber angle discontinuities found in the pulmonary vein antra (Narayan et al., 2008; Pashakhanloo et al., 2016) or crista terminalis (Gonzales et al., 2014), creating anisotropic conduction which may lead to reentry.

## Strategies to Attenuate and Risk-Stratify Atrial Fibrillation Prior to Ablation

### Risk Factor Modification

Recent studies have shown that risk factor modification can reduce or suppress AF (Pathak et al., 2014, 2015; Lau et al., 2017). Obstructive sleep apnea (OSA) may lead to atrial electrophysiologic remodeling with increased atrial fibrosis and downregulation of connexin-43 due to repeated apneic episodes (Iwasaki et al., 2014). In a rat model, this resulted in substantial atrial conduction slowing and increased inducibility and duration of AF. Patients with OSA undergoing clinical AF ablation were found to have a reduction in bi-atrial voltage, widespread conduction abnormalities and longer sinus node recovery times (Dimitri et al., 2012).

Multiple studies have demonstrated the strong link between obesity and risk of developing AF. In sheep models, those with more significant obesity were found to have increased cytoplasmic transforming growth factor β1, platelet-derived growth factor, and larger left atrial size (Mahajan et al., 2015). Furthermore, there was also increased atrial fibrosis, infiltration of the epicardial fat into the posterior left atrial wall, heterogenous and slowed atrial conduction velocity, and higher rates of inducible and spontaneous AF in the obese group in both sheep and humans (Munger et al., 2012; Abed et al., 2013). Weight reduction is associated with improved AF control. In the LEGACY study, weight loss of ≥10% resulted in a 6-fold increased probability of arrhythmia-free survival (Pathak et al., 2015). Similarly, the ARREST-AF study showed that weight reduction with other risk factor modifications resulted in longer arrhythmia-free survival after AF ablation (Pathak et al., 2014). These studies suggest that atrial remodeling associated with obesity may be reversible with weight reduction (Aldaas et al., 2019).

Varying degrees of alcohol consumption has been associated with risk of incident AF and recurrence of AF after catheter ablation. This may be partly contributed by alcohol’s association with other known risk factors for AF such as obesity, hypertension, and disordered sleep pattern. However, prior work has shown acute changes in atrial electrophysiology as a direct result of alcohol consumption and binge drinking, including shortening of the effective refractory period, slowed intra-atrial conduction, and prolonged p wave duration (Voskoboinik et al., 2016). Additionally, chronic drinking is an independent multivariate predictor of discrete atrial fibrosis (Qiao et al., 2015). Regarding the effect of alcohol cessation on burden of AF, the ARREST-AF study demonstrated decreased AF recurrence and symptom severity in patients with risk factor management including decreased alcohol consumption (Pathak et al., 2014).

### Identify Fibrosis

Atrial fibrosis can be identified with a range of imaging and mapping technologies, of which some are detailed in a 2016 EHRA consensus statement (Donal et al., 2016). Table 1 lists techniques that have been developed to characterize atrial substrate. However, it is important to note that all modalities identify indirect surrogates for atrial fibrosis, and use of fibrosis to guide ablation may be non-specific and as fibrosis is not synonymous with arrhythmogenicity.

**TABLE 1:** Clinical technologies to localize areas of fibrosis.

**Technique**	**Advantages**	**Disadvantages**	**Landmark clinical studies**
Cardiac MRI	Non-invasive, high signal-to-noise ratio	Artifact from cardiac devices, reproducibility of segmentation/windowing	DECAAF, Marrouche et al., 2014
Perfusion CT	High resolution, quick	Radiation, contrast	Ling et al., 2015
MRI-based computer modeling	Non-invasive, functional data in addition to structural data	Assumptions in computational model, variabilities with segmentation	Boyle et al., 2019
Electro-anatomic voltage mapping	High resolution, real-time	Invasive, time-intensive, assumptions of electrode recordings	Jadidi et al., 2016; Kottkamp et al., 2016; Yagishita et al., 2017

#### Cardiac MRI

**FIGURE 3:**
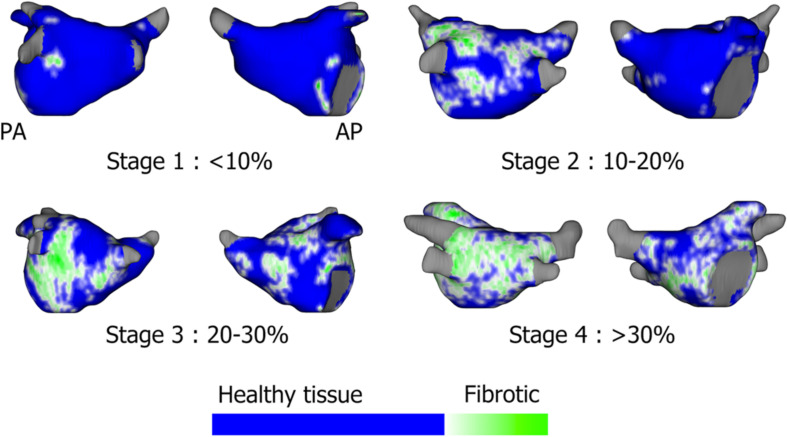
Assessment of left atrial fibrosis burden using segmented gadolinium-enhanced MRI scans based on the Utah stages: Utah 1: <10%, Stage 2: 10–20%, Stage 3: 20–30%, Stage 4: >30% fibrotic tissue. Sohns and Marrouche (2020), reprinted with permission.

In some centers, cardiac MRI is used pre-procedurally to characterize atrial anatomy and burden of fibrosis in preparation for catheter ablation (Mahnkopf et al., 2010; Daccarett et al., 2011; Malcolme-Lawes et al., 2013; McGann et al., 2014; Calkins et al., 2017; Sohns and Marrouche, 2020). The multicenter prospective DECAAF trial (Marrouche et al., 2014) has established the role of MRI as a prognostic tool to predict success of PVI ablation based on the degree of atrial fibrosis (Figure 3). Additionally, other studies have evaluated the ability of MRI to detect gaps in PVI lesions in order to identify PV reconnections (Bisbal et al., 2014). However, the utility of MRI to localize fibrotic areas as potential targets for ablation is still unknown and is under investigation with the DECAAF-2 trial (NCT 02529319). A limitation of current cardiac MRI technology is artifact from cardiac devices and variability in acquisition sequences and thresholding (Karim et al., 2013; Calkins et al., 2017). A second challenge is that fibrotic areas may not always correlate with arrhythmogenicity. Additional work is required to determine the optimal role of MRI in AF procedural planning.

##### MRI based computer simulations

One proposed method to identify areas of fibrosis with potential arrhythmogenicity is building computer simulations using fibrotic areas derived from MRI combined with electroanatomic computer simulations to identify re-entrant drivers. This technique was described in a proof-of-concept clinical study showing feasibility of this non-invasive method (Zahid et al., 2016; Boyle et al., 2019). Prospective studies are needed to see whether this technique may improve AF ablation outcomes.

##### Cardiac CT

A proposed technique using perfusion cardiac computed tomography (CT) to identify surrogate regions of fibrosis was described by Ling and colleagues (Ling et al., 2015). In this technique, contrast-enhanced gated cardiac CT were segmented by degree of contrast attenuation, and areas of low attenuation correlated with low voltage points obtained from invasive electroanatomic bipolar voltage maps (*p* = 0.04) and qualitative agreement in about 80% of patients. Contemporary cardiac CT provides improved resolution (∼0.5 mm) as compared to MRI (∼1.5–2.0 mm) (Saeed et al., 2015), but has a lower signal-to-noise ratio. Although cardiac CT imaging is routinely obtained for evaluation of gross atrial anatomy prior to AF ablation, additional work is needed to both improve the ability of CT perfusion imaging to detect fibrosis and define its role in targeting AF-sustaining substrate.

##### Electro-Anatomic mapping

Invasive electroanatomic mapping is an established method of characterizing electrical substrate to identify regions as a surrogate for fibrosis. Improvements in electroanatomic mapping systems and multi-electrode mapping catheters have enabled spatial resolution down to 2–3 mm and higher density maps. Similar to substrate homogenization-based strategies to ablate ventricular tachycardia, individually tailored substrate ablation has been proposed and studied for AF. However, unlike ventricular tachycardia in which substrate mapping can be used to define the critical isthmus of a scar-based monomorphic VT circuit, the precise fibrotic microstructure underlying AF is less well characterized.

The efficacy of a substrate modification approach utilizing box isolation of fibrotic areas (BIFA) was tested in 28 patients with both paroxysmal and persistent AF who had low voltage areas <0.5 mV identified using a contact force ablation catheter (Kottkamp et al., 2016). The BIFA approach entails surrounding low voltage areas with linear ablation lines anchored to non-conducting regions such as wide area circumferential (WACA) ablation circles. This resulted in a 90% 1 year freedom in 10 paroxysmal AF patients and 72% 1 year freedom in 18 persistent AF patients. In another study by Kircher et al. (2018), 124 patients with either paroxysmal or persistent AF undergoing PVI were randomized to additional standard linear ablation versus an individually tailored approach to target low voltage areas. Voltage mapping was performed using a circular mapping catheter (1 mm electrode spacing) with low voltage cut-offs of <0.5 mV. Ablation of these voltage areas either in a cluster, linear ablation anchored to non-excitable regions or box isolation resulted in a significant improvement in freedom from atrial arrhythmia (68 vs 42%) after a single procedure. Although there was an improvement in this technique over empiric linear ablation, the recurrence in almost a third of patients suggests that the underlying etiology of AF in these patients still has not been fully addressed. Disadvantages to substrate homogenization include the non-specificity of low voltage zones without integrated functional data and limitations of invasive voltage mapping described below.

Several clinical studies have attempted to characterize voltage cut-offs to represent the spectrum of atrial fibrosis (Table 2). In general, these studies used either ablation catheters with 4 mm electrodes (Oakes et al., 2009; Spragg et al., 2012) or multi-electrode catheters (Verma et al., 2005; Kapa et al., 2014; Rolf et al., 2014; Anter et al., 2015; Kottkamp et al., 2016) with 1 mm electrodes and 2–6 mm electrode spacing to define: dense scar at <0.2 mV, borderzone fibrosis at <0.5 mV and normal tissue >0.5 mV when compared in AF patients with healthy or abnormal atria, by the presence of LGE on MRI in 4 studies (Oakes et al., 2009; Spragg et al., 2012; Kapa et al., 2014; Anter et al., 2015). However, some investigators have argued abnormal tissue can exist with voltages <1.5 mV and viable tissue exists at >0.05 mV. This discrepancy could be contributed by several limitations and misconceptions of interpreting low voltage as a surrogate for fibrosis as described by Josephson and Anter (2015). Electrogram voltage may be affected by conduction velocity, fiber orientation and curvature, relationship of fiber orientation to the propagating wavefront, tissue contact, edema, fat and characteristics of the recording catheter such as electrode size, interelectrode spacing. Newer multielectrode catheters have been developed which could potentially address some of these issues, such as the orthogonal grid catheters and high-density baskets with 0.4 mm electrodes; however, these have not been systematically studied in this regard.

**TABLE 2:** Studies of bipolar voltage cutoffs.

**Voltage cut-off (mV)**	**Study**	**Mapping catheter used**	**Gold standard for fibrosis**	**Patients**	**Rhythm**
<0.2: dense scar <0.5: diseased	Verma et al., 2005	Circular (2-6-2 mm spacing)	Clinical history	700	NSR
<0.1: dense scar >0.5: normal	Oakes et al., 2009	Linear (4 mm tip, 1-7-4 mm)	LGE-MRI	54	60% NSR
>0.5 mV: normal	Spragg et al., 2012	Linear (3.5 mm tip, 2 mm)	LGE-MRI	10	?
<0.27: scar >0.45: normal	Kapa et al., 2014	Circular (2-6-2 mm)	LGE-MRI	20	NSR
<0.2: scar >0.5: normal	Rolf et al., 2014	Circular (2-6-2 mm or 2-4-2 mm)	Clinical history	178	NSR
<0.25: dense scar >0.48: normal	Anter et al., 2015	Multi-spline (2-6-2 mm)	LGE-MRI	30	NSR
<0.5: scar >1.5: normal	Kottkamp et al., 2016	Circular (2-6-2 mm)	Clinical history	41	NSR

*NSR, normal sinus rhythm; AF, atrial fibrillation.*

### Identifying AF Triggers

Patients who have a high burden of atrial flutter or supraventricular tachycardias (SVT) may undergo atrial remodeling and develop both electrical and fibrotic substrate for AF (Franz et al., 1997). Thus, identifying a defined, consistent trigger is an important component of an AF ablation strategy (Calkins et al., 2017).

#### Supraventricular Tachycardias and Atrial Tachycardia/Flutters

Prior studies have shown an association between AF and supraventricular tachycardia (SVT) (Sauer et al., 2006) and cavo-tricuspid isthmus dependent atrial flutters (TAFL) (Pérez et al., 2009). Based upon these studies, it is recommended to evaluate for co-existing SVT or atrial tachycardia/flutter mechanisms either before or after AF ablation.

#### Pulmonary Vein Triggers

Foundational work in AF ablation demonstrated the importance of pulmonary vein (PV) triggers to AF (Haïssaguerre et al., 1998). The initial approach to PV trigger ablation was to perform segmental pulmonary vein isolation. A subsequent randomized study by Arentz et al. (2007) demonstrated improved outcomes with wide area circumferential ablation (WACA) compared to segmental ablation, potentially by disrupting other sustaining AF mechanisms at the PV antra characterized by fiber angle discontinuities (Pashakhanloo et al., 2016) and increased repolarization restitution (Narayan et al., 2008).

#### Non-PV Triggers

Ongoing work has revealed that non-PV ectopic beats and PACs may be present in 10–33% of patients with AF (Chen and Tai, 2005; Takigawa et al., 2015; Santangeli et al., 2016; Hojo et al., 2017), and suggested the potential utility of aggressive trigger induction with high dose isoproterenol. These triggers may originate from the posterior LA wall, superior vena cava (SVC), crista terminalis, coronary sinus (CS), Eustachian ridge, Ligament of Marshall, and left atrial appendage (Lin et al., 2003; Chen and Tai, 2005; Calkins et al., 2017). Additional work is required to determine the significance of these triggers to perpetuation of AF and whether this approach yield long-term improvement in AF-free survival after ablation.

### Identify Drivers

A challenge in mapping drivers of AF is that standard activation mapping techniques using point by point mapping are unable to fully resolve the evolving wavefront propagation patterns during AF due to lack of having a standard reference. A second challenge is that AF drivers potentially utilize several sites of abnormal substrate during ongoing AF.

To address these challenges, an increasing number of specialized electrogram processing techniques and panoramic mapping methods have been developed. The variety of technologies, their requirements, risks and available data are illustrated in Table 3.

**TABLE 3:** Clinical technologies to Localize AF drivers.

**Technique**	**First clinical study**	**Methodology**	**Equipment needed**	**Access**	**Disadvantages**
FIRM	2012	Phase mapping	64-electrode basket	8.5 Fr sheath	Basket catheter tissue contact, false-positive rotors
ECGi	2014	Activation and phase mapping	252-electrode disposable vest	Non-invasive	Epicardial potentials only/misses septal sites
Localized Electrogram Organization	2016	Activation, voltage & EGM characteristics	Multi-electrode catheter	8.5 Fr sheath	Subjective qualititative EGM assessment, non-panoramic
CARTOFINDER	2018	Activation mapping	64-electrode basket	8.5 Fr sheath	Basket catheter tissue contact, activation mapping limited by low temporo-spatial resolution
Acutus	2019	Activation mapping	48 electrode basket	16 Fr sheath	Large 16 Fr trans-septal sheath risks
Wavefront Mapping	2019	Propagation vector mapping	64-electrode basket	8.5 Fr sheath	Basket catheter tissue contact
FAST	2020	Spectral and unipolar EGM	Multi-electrode catheter	8.5 Fr sheath	Non-panoramic method, time consuming

### Focal Impulse and Rotor Modulation

One of the first technologies developed to specifically target the sustaining mechanisms of AF was focal impulse and rotor modulation (FIRM) mapping (Narayan et al., 2012a,b,c, 2013). This technique utilizes 64-electrode basket catheter recordings during AF analyzed by computational activation and phase analysis to identify semi-stable focal (centripetal activation) and rotational activation patterns. The results from the initial trial of this approach were reported in the CONFIRM trial (Narayan et al., 2012d), in which 92 patients undergoing AF ablation underwent pulmonary vein isolation alone versus PVI plus rotor ablation. Patients undergoing PVI + FIRM ablation experienced a greater number of AF terminations during ablation and had a greater AF-free survival at a median of 273 days after ablation. Subsequent work demonstrated that these results were durable, improving AF-free survival over a median follow-up of 870 days (Narayan et al., 2014). These outcomes were reproduced in independent studies from more than 10 sites (Miller et al., 2014) including Miller et al. (2017) who reported their results for 170 consecutive patients undergoing AF ablation employing PVI plus AF rotor ablation. Freedom from all atrial arrhythmias was 75% in patients with persistent AF and 57% in longstanding persistent AF at 1 year, off antiarrhythmic drug therapy. A meta-analysis by Baykaner et al. (2018) analyzed all published studies of FIRM mapping and ablation found a significant improvement in freedom from atrial arrhythmia recurrence in patients undergoing pulmonary vein isolation plus FIRM ablation. Ongoing work is required to determine the precise population who maximally benefit from this approach.

### Electrocardiographic Imaging

**FIGURE 4:**
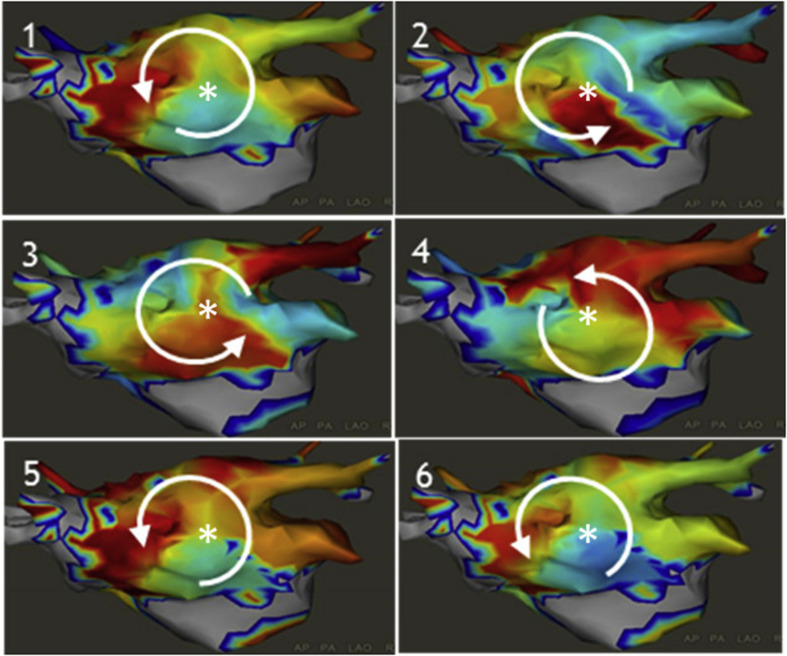
Examples of an atrial rotor **(A)** and a focal driver **(C)** which were identified using the inverse solution computer modeling and phase mapping of body surface epicardial potentials **(B)**. Knecht et al. (2017), reprinted with permission.

Electrocardiographic imaging (ECGVue, Medtronic, Minneapolis, MN) is a non-invasive technique utilizing a 252-electrode vest integrated with a non-contrast CT has been used to record unipolar epicardial potentials during AF using inverse solution modeling (Ramanathan et al., 2004). Similarly, phase mapping has been applied to these potentials (Haissaguerre et al., 2014), and in a study of 103 patients with persistent AF, identified rotational and focal drivers (Figure 4).

Although changing wavefronts and transient reentrant activity were observed, AF drivers occurred repetitively in the defined regions. Ablation of such regions terminated persistent AF in 75% of patients and resulted in 1 year freedom from AF in 85% of patients. In the AFACART study (Knecht et al., 2017) of 118 patients with persistent AF, a step-wise ablation approach (driver only then PVI then linear ablation) showed that driver-only ablation terminated AF in 64% of patients, and this step-wise approach resulted in single procedure 1 year freedom from AF in 78% of patients, though 49% experienced atrial tachycardia.

### Dipole Density Mapping

**FIGURE 5:**
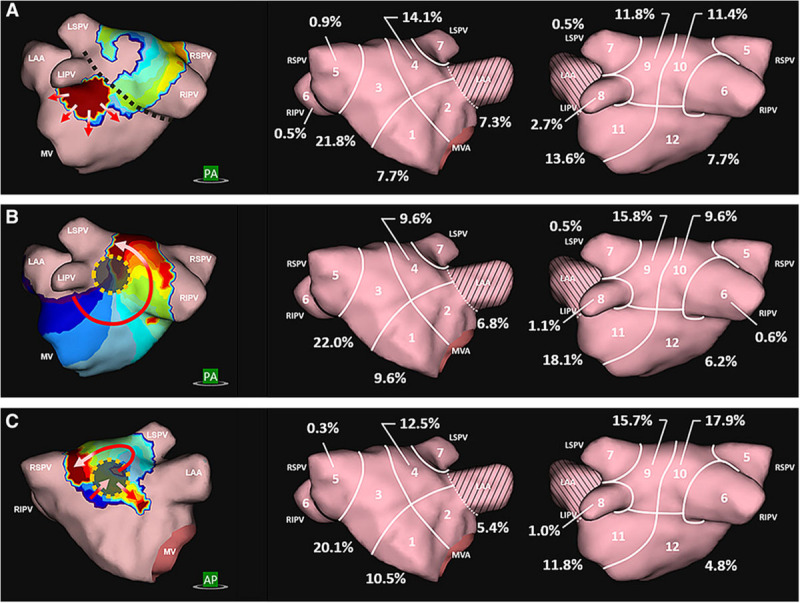
Dipole density mapping reveals 3 distinct activation patterns: **(A)** focal activity originating from the mitral isthmus, **(B)** rotational activity originating from the posterior wall, and **(C)** localized irregular activation characterized by repetitive multidirectional entry, exit, and pivoting through a fixed site. Willems et al. (2019), reprinted with permission.

A system using dipole density mapping combined with ultrasound (Grace et al., 2017; Shi et al., 2020) (AcQMap, Acutus Medical Inc., Carlsbad, CA, United States) has been developed to create high resolution endocardial activation maps (150,000 points per second). AcQMap consists of a basket catheter with 48 ultrasound transducers and electrodes to sample the intracardiac potential field to create an instantaneous activation map using a non-contact, inverse solution algorithm (Figure 5). This basket is placed via a 16 French steerable sheath.

The AcQMap system was validated with contact mapping in 20 patients (Shi et al., 2020) showing good agreement for points up to 4 cm away from the center of the catheter. It was prospectively studied in 127 patients with persistent AF in the multicenter UNCOVER-AF trial (Willems et al., 2019) and identified organized sources including localized irregular activation (repetitive conduction through a confined zone, Figure 5C), focal sources and rotational activation were found with an average of 5 sources per patient. Ablation of these sources resulted in termination in 32% of patients and 1-year freedom from AF in 73% with a single procedure and 93% with a second procedure. Randomized studies are still needed to establish a clear benefit using this strategy.

### Localized Electrogram Dispersion

**FIGURE 6:**
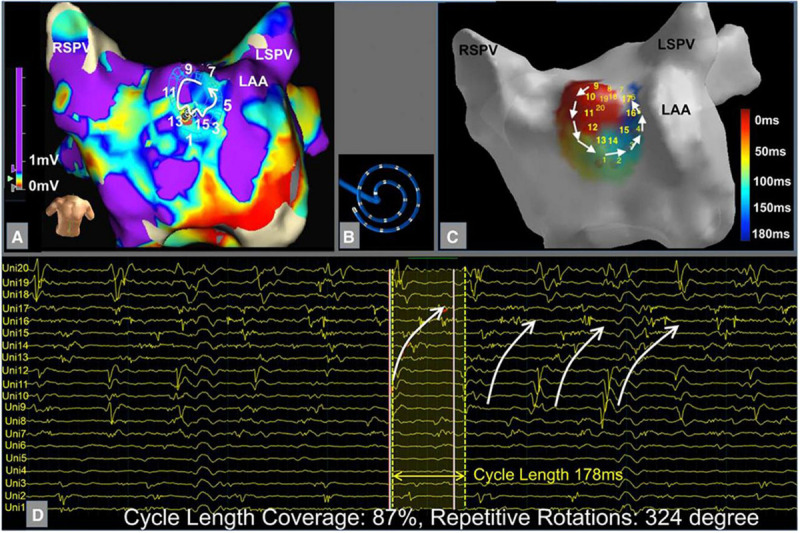
Example of localized electrogram dispersion. **(A)** Bipolar voltage map with site of rotational activity. **(B)** Orientation of the multielectrode circular mapping catheter. **(C)** Activation sequence of rotational activity where ablation terminated atrial fibrillation. **(D)** Corresponding unipolar electrograms show repetitive rotational activation sequence. Jadidi et al. (2016) reprinted with permission.

A few methods have been proposed using qualitative analysis of electrogram temporospatial organization obtained from standard circular or multi-spline catheters. These methods expand upon complex fractionated atrial electrogram (CFAE) mapping by further evaluating propagation of electrograms on neighboring electrodes in an organized fashion to determine the presence of an active driver. Jadidi et al. (2016) proposed a method to identify regions with low voltage (<0.5 mV, Figure 6A) and electrograms spanning >70% of the AF cycle length over neighboring electrodes (Figures 6B,C) to identify a surrogate of an AF driver.

In a prospective study of 62 patients with persistent AF, ablation of these low voltage areas in addition to PVI led to acute AF termination in 73% and single procedure 1 year freedom from AF in 69%, compared to 47% in a matched PVI only control group (*p* < 0.001).

Another method described by Seitz et al. (2017) involves localization of regions of temporal and spatial dispersion of electrograms (minimum of 3 adjacent bipoles with activation spanning the entire AF cycle length) using a multi-spline catheter (Pentaray, Biosense-Webster, CA, United States). In the SUBSTRATE HD study (Seitz et al., 2017) of 105 patients (77% persistent AF), ablation of only driver regions terminated AF in 95% of patients, and 1.5-year freedom from atrial arrhythmias (median 1.4 procedures) was 85% compared to 59% of a validation control group who underwent step-wise PVI and linear ablation approach. Further randomized and multicenter centers with inexperienced operators are needed to confirm these promising results for localized driver ablation.

### CARTOFINDER^TM^

**FIGURE 7:**
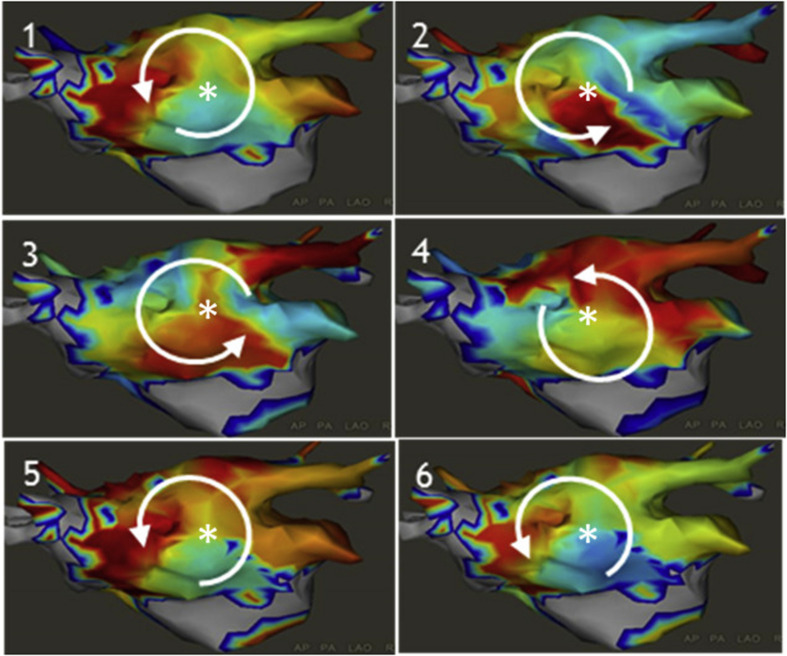
Example of CARTOFINDER showing a counter clockwise rotational repetitive activation pattern through time (panels 1–6). Daoud et al. (2017) reprinted with permission.

The CARTOFINDER^TM^ system (Daoud et al., 2017) is an activation mapping based algorithm that records unipolar endocardial electrograms from 64-electrode basket catheters (Figure 7, left panel). The system calculates the percentage of the atrial surface geometry that is covered by the basket to guide repositioning. Activation patterns are then analyzed on the CARTO system to identify focal or reentrant drivers (Figures 7A–D).

In a study of 20 patients with persistent AF, CARTOFINDER identified rotational or focal drivers in all patients and AF terminated in half of the patients with driver ablation (Honarbakhsh et al., 2018). Randomized, longer-term outcome studies are needed to determine whether this method may effectively identify drivers to improve freedom from AF.

### Focal Source and Trigger Mapping (FaST)

**FIGURE 8:**
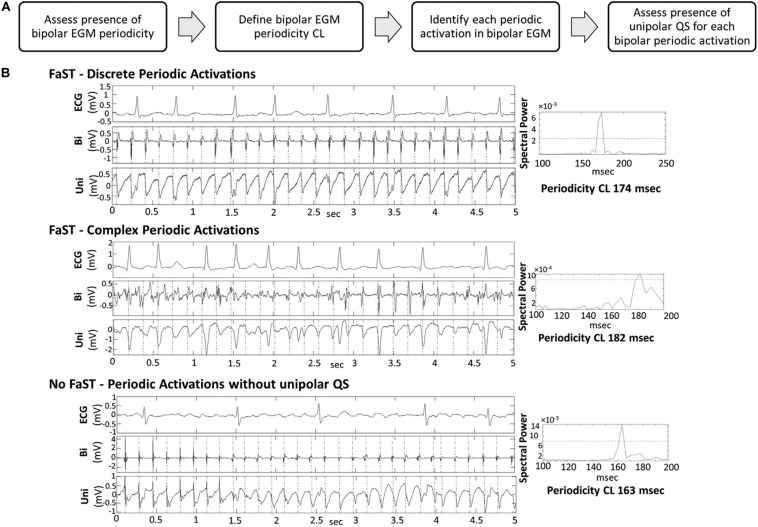
**(A)** Workflow of algorithm in the FaST system. **(B)** Periodic activation of electrograms during AF and spectral analysis. Chauhan et al. (2020) reprinted with permission.

A novel quantitative algorithm (Gizurarson et al., 2016; Daoud et al., 2017) identifying sites with periodicity and QS unipolar electrogram morphology was described by Chauhan et al. (2020). From bipolar and unipolar electrograms collected from a circular mapping electrode (Lasso, Biosense-Webster), the electrograms are analyzed by an algorithm (Figure 8A) that assesses bipolar EGM periodicity (segments with a spectral peak >10% of the total spectral power) and assesses the presence of QS morphology on the unipolar EGMs as a surrogate for organized focal drivers (Figure 8B).

This algorithm was tested in a randomized study of 80 patients (Chauhan et al., 2020) (48% persistent AF) to PVI + FAST versus PVI only, and resulted in a trend toward improved 1-year freedom from AF (74% with PVI + FAST compared to 51% with PVI-only, *p* = 0.06). Larger multicenter trials are needed to see if this method may significantly improve AF ablation success.

### Wavefront Field Mapping

**FIGURE 9:**
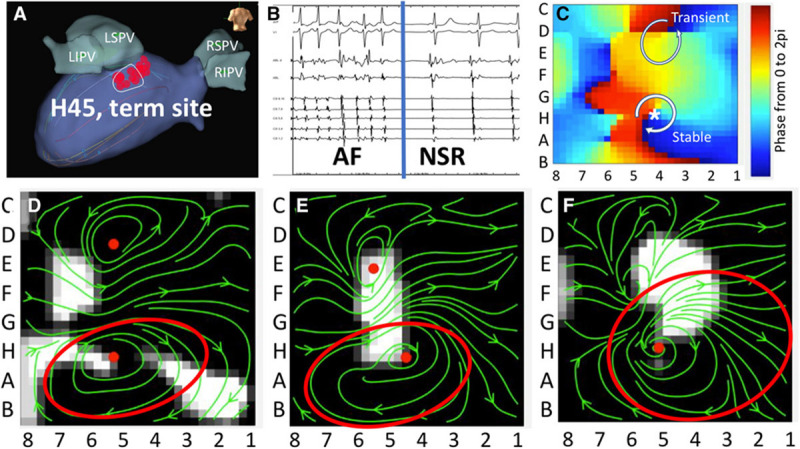
**(A)** Targeted ablation of a rotor in the left posterior left atrium resulting in panel **(B)** AF termination in a patient with clinical AF. Activation times are converted to phase analysis **(C)**. **(D–F)** Vector fields of potential AF driver during AF. Leef et al. (2019) reprinted with permission.

A promising technique was recently proposed using wavefront field mapping (Leef et al., 2019) to reveal organized areas of control during AF (Figures 9A,B). This novel vector mapping method computes activation times to calculate phase (Figure 9C), activation fronts, and gradient matching to display the vector fields (Figures 9D–F) to describe propagation of these fronts.

An advantage of this method is the ability to identify regions that control larger areas of the atria during AF, and thus distinguish true driver of AF compared to passive organized areas. This concept was studied retrospectively in 54 patients (Leef et al., 2019) in whom ablation of proposed drivers (from phase mapping) terminated AF, and all sites that were found to control larger atrial areas terminated AF with ablation. Prospective studies are ongoing to evaluate the ability of this method to identify critical drivers of AF to improve the targeted therapy of AF.

## What Is the Optimal Ablation Strategy to Target Mechanisms of AF?

The plethora of new imaging and mapping technologies reviewed above attempt to provide a personalized approach to improve the treatment of AF. Although there is a strong link between atrial fibrosis and electrical drivers (Cochet et al., 2018; Hansen et al., 2018), they do not always co-exist (Chrispin et al., 2016; Schade et al., 2016), suggesting electrical remodeling and structural heterogeneities other than fibrosis are also important AF mechanisms (Stiles et al., 2009; Lalani et al., 2012; Schricker et al., 2014; Zaman and Narayan, 2015). There are some patients who may have only an electrical substrate without fibrosis (such as lone AF due to a pulmonary vein driver), and there are other patients who have extensive atrial fibrosis with multiple AF drivers (such as a patient with familial atrial cardiomyopathy). In the first case, PVI alone or driver mapping-guided ablation may be enough to eliminate the AF mechanism, but may not be enough in the second case. While meta-analysis of driver ablation studies show a benefit toward driver ablation (Baykaner et al., 2018), AF recurrence still recurs in ∼30%. This may be partly due to technological shortcomings and operator inexperience, but a possibility is that new drivers may recur in certain patients with progressive primary atrial cardiomyopathies.

More work is needed to determine how to identify patients with progressive underlying atrial cardiomyopathies and how to incorporate a substrate modification strategies in addition to AF drivers. However, importantly, more ablation is not necessarily better particularly with empiric atrial debulking strategies, as STAR-AF2 showed a proarrhythmic effect of empiric linear ablation primarily due to creation of substrate for atypical atrial flutters (Verma et al., 2015) and development of stiff atrial syndrome (Gibson et al., 2011).

**FIGURE 10:**
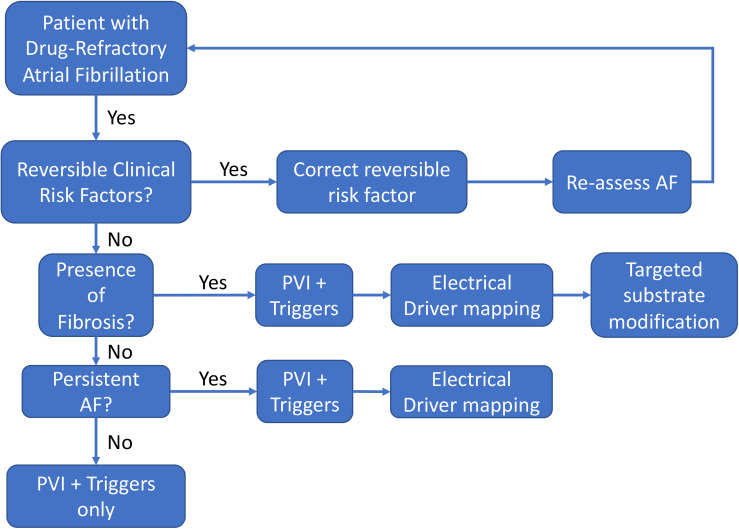
Flowchart of a decision-support strategy regarding the use of supplemental AF mapping and targeting technologies.

Based upon the above discussion, we propose the following strategy for the management of drug-refractory AF (Figure 10), incorporating an organized approach to manage and reduce risk factors, and to use imaging to reveal the diverse types of AF. First, reversible clinical risk factors, such as obesity, hypertension, obstructive sleep apnea, and excessive alcohol use should be optimized in all patients to help reverse atrial remodeling (Lau et al., 2017). Second, PVI should usually be performed in all patients as an initial strategy. In the most recent 2017 expert

consensus document for AF ablation, pulmonary vein isolation is the only ablation strategy given a Class 1 indication (Calkins et al., 2017). Third, elimination of AF triggers such as frequent PACs/atrial tachycardias should also be strongly considered, which is given a Class 2A indication. Fourth, in patients in whom AF sustains or remains inducible, adjunctive strategies addressing patient-specific AF mechanisms may be needed to improve the success of ablation. To address the potential for fibrotic mechanisms discussed previously, pre-procedural imaging or comprehensive invasive voltage mapping should be considered to determine the presence of significant atrial fibrosis which may prognosticate a need to perform electrical driver ablation and/or substrate modification to eliminate extra-PV AF mechanisms. Finally, in patients with persistent AF in whom AF is more likely to recur after PVI alone, electrical driver mapping should be considered to eliminate extra pulmonary vein AF mechanisms in a targeted, patient-specific approach. At present, the choice of adjunction functional mapping should in part be determined by operator experience and electrophysiology laboratory factors in the absence of definitive clinical trial data evaluating the new adjunctive technologies. While the current AF ablation consensus guidelines do discuss some of the earlier imaging and driver technologies, they are given a Class IIb indication due to lack of good quality clinical data. With improvements in mapping and imaging technologies discussed above, we await clinical trials to confirm the optimistic preliminary clinical studies supporting these mechanistic AF treatment strategies.

## Conclusion

This review summarizes data linking fibrotic atrial cardiomyopathy with AF drivers and summarizes current clinical approaches for the targeted therapy of substrate and electrical mechanisms underlying atrial fibrillation. As the two patients presented at the beginning demonstrate, causes of AF may be heterogeneous; it is possible that AF in patient 1 may have been caused by electrical substrate only (triggered activity from an ectopic pulmonary vein source). In contrast, patient 2 may have an underlying primary atrial cardiomyopathy, compounded by the presence of scar from prior ablation, and although driver ablation terminated AF, he may have recurrence of AF with new driver sites. Similar to scar-based VT, a combination of substrate modification guided by electrical driver mapping may be needed in certain patients.

We believe that current data support an approach in which risk factor modification is addressed in all patients, and a patient specific strategy incorporating targeted therapy of structural and electrical substrate is considered in particular for patients with anatomic and functional remodeling suggestive of advanced atrial remodeling and AF drivers outside the pulmonary vein anatomy. Ongoing work is required to determine the optimal combination of imaging and AF functional mapping to optimize procedural results in the management of patients with refractory arrhythmias.

## Disclosures

Dr. Ho receives grant support from the American Heart Association (AHA 19CDA34760021), National Institutes of Health (NIH 1KL2TR001444-06), and reports equity in Vektor Medical Inc. unrelated to this work. Dr. Krummen receives grant support from the UCSD Galvanizing Engineering in Medicine Foundation. He also reports equity in Vektor Medical unrelated to this work.

## Author Contributions

GH reviewed the current literature for the present manuscript, wrote the outline, identified important figures, composed the manuscript, and provided critical editing of the manuscript. AL performed background research for the manuscript and provided critical editing of the manuscript. DK reviewed the literature, composed the manuscript, and provided critical editing of the manuscript. All authors contributed to the article and approved the submitted version.

## Conflict of Interest

The authors declare that the research was conducted in the absence of any commercial or financial relationships that could be construed as a potential conflict of interest.

## References

[B1] Abed H. S. Samuel C. S. Lau D. H. Kelly D. J. Royce S. G. Alasady M. (2013). Obesity results in progressive atrial structural and electrical remodeling: implications for atrial fibrillation. *Hear. Rhythm* 10 90–100. 10.1016/j.hrthm.2012.08.043 23063864

[B2] Aldaas O. M. Lupercio F. Han F. T. Hoffmayer K. S. Krummen D. Ho G. (2019). Meta-analysis of effect of modest (=10%) weight loss in management of overweight and obese patients with atrial fibrillation. *Am. J. Cardiol.* 124 1568–1574. 10.1016/j.amjcard.2019.08.009 31540665 PMC7089802

[B3] Angel N. Li L. MacLeod R. S. Marrouche N. Ranjan R. Dosdall D. J. (2015). Diverse fibrosis architecture and premature stimulation facilitate initiation of reentrant activity following chronic atrial fibrillation. *J. Cardiovasc. Electrophysiol.* 26 1352–1360. 10.1111/jce.12773 26249367 PMC4729779

[B4] Anter E. Tschabrunn C. M. Josephson M. E. (2015). High-resolution mapping of scar-related atrial arrhythmias using smaller electrodes with closer interelectrode spacing. *Circ. Arrhythmia Electrophysiol.* 8 537–545. 10.1161/circep.114.002737 25792508

[B5] Arentz T. Weber R. Bürkle G. Herrera C. Blum T. Stockinger J. (2007). Clinical perspective. *Circulation* 115 3057–3063. 17562956 10.1161/CIRCULATIONAHA.107.690578

[B6] Baykaner T. Lalani G. G. Schricker A. Krummen D. E. Narayan S. M. (2014). Mapping and ablating stable sources for atrial fibrillation: summary of the literature on Focal Impulse and Rotor Modulation (FIRM). *J. Interv. Card Electrophysiol.* 40 237–244. 10.1007/s10840-014-9889-8 24647673

[B7] Baykaner T. Rogers A. J. Meckler G. L. Zaman J. Navara R. Rodrigo M. (2018). Clinical implications of ablation of drivers for atrial fibrillation: a systematic review and meta-analysis. *Circ. Arrhythm. Electrophysiol.* 11:e006119. 10.1161/CIRCEP.117.006119 PMC6474343 29743170

[B8] Bisbal F. Guiu E. Cabanas-Grandío P. Berruezo A. Prat-Gonzalez S. Vidal B. (2014). CMR-guided approach to localize and ablate gaps in repeat AF ablation procedure. *JACC Cardiovasc. Imaging* 7 653–663. 10.1016/j.jcmg.2014.01.014 24813966

[B9] Boyle P. M. Zghaib T. Zahid S. Ali R. L. Deng D. Franceschi W. H. (2019). Computationally guided personalized targeted ablation of persistent atrial fibrillation. *Nat. Biomed. Eng.* 3 870–879. 31427780 10.1038/s41551-019-0437-9 PMC6842421

[B10] Caballero R. De La Fuente M. G. Gómez R. Barana A. Amorós I. Dolz-Gaitón P. (2010). In humans, chronic atrial fibrillation decreases the transient outward current and ultrarapid component of the delayed rectifier current differentially on each atria and increases the slow component of the delayed rectifier current in both. *J. Am. Coll. Cardiol.* 55 2346–2354. 10.1016/j.jacc.2010.02.028 20488306

[B11] Calkins H. Hindricks G. Cappato R. Kim Y. H. Saad E. B. Aguinaga L. (2017). 2017 HRS/EHRA/ECAS/APHRS/SOLAECE expert consensus statement on catheter and surgical ablation of atrial fibrillation. *Hear. Rhythm* 14 e275–e444. 10.1016/j.hrthm.2017.05.012 PMC6019327 28506916

[B12] Chang S. Tai C. Lin Y. Wongcharoen W. Lo L. Tuan T. (2007). Biatrial substrate properties in patients with atrial fibrillation. *J. Cardiovasc. Electrophysiol.* 18 1134–1139. 10.1111/j.1540-8167.2007.00941.x 17764448

[B13] Chauhan V. S. Verma A. Nayyar S. Timmerman N. Tomlinson G. Porta-Sanchez A. (2020). Focal source and trigger mapping in atrial fibrillation: randomized controlled trial evaluating a novel adjunctive ablation strategy. *Hear. Rhythm* 17 683–691. 10.1016/j.hrthm.2019.12.011 31991116

[B14] Chen S. A. Tai C. T. (2005). Catheter ablation of atrial fibrillation originating from the non-pulmonary vein foci. *J. Cardiovasc. Electrophysiol.* 16 229–232. 10.1046/j.1540-8167.2005.40665.x 15720466

[B15] Chou C. C. Nihei M. Zhou S. Tan A. Kawase A. Macias E. S. (2005). Intracellular calcium dynamics and anisotropic reentry in isolated canine pulmonary veins and left atrium. *Circulation* 111 2889–2897. 10.1161/circulationaha.104.498758 15927973

[B16] Chrispin J. Ipek E. G. Zahid S. Prakosa A. Habibi M. Spragg D. (2016). Lack of regional association between atrial late gadolinium enhancement on cardiac magnetic resonance and atrial fibrillation rotors. *Hear. Rhythm* 13 654–660. 10.1016/j.hrthm.2015.11.011 26569460

[B17] Chugh S. S. Havmoeller R. Narayanan K. Singh D. Rienstra M. Benjamin E. J. (2014). Worldwide epidemiology of atrial fibrillation: a global burden of disease 2010 study. *Circulation* 129 837–847. 10.1161/circulationaha.113.005119 24345399 PMC4151302

[B18] Cochet H. Dubois R. Yamashita S. Al Jefairi N. Berte B. Sellal J. M. (2018). Relationship between fibrosis detected on late gadolinium-enhanced cardiac magnetic resonance and re-entrant activity assessed with electrocardiographic imaging in human persistent atrial fibrillation. *JACC Clin. Electrophysiol.* 4 17–29. 10.1016/j.jacep.2017.07.019 29479568 PMC5824731

[B19] Csepe T. A. Hansen B. J. Fedorov V. V. (2017). Atrial fibrillation driver mechanisms: insight from the isolated human heart. *Trends Cardiovasc. Med.* 27 1–11. 10.1016/j.tcm.2016.05.008 27492815 PMC5122472

[B20] Daccarett M. Badger T. J. Akoum N. Burgon N. S. Mahnkopf C. Vergara G. (2011). Association of left atrial fibrosis detected by delayed-enhancement magnetic resonance imaging and the risk of stroke in patients with atrial fibrillation. *J. Am. Coll. Cardiol.* 57 831–838. 10.1016/j.jacc.2010.09.049 21310320 PMC3124509

[B21] Daoud E. G. Zeidan Z. Hummel J. D. Weiss R. Houmsse M. Augostini R. (2017). Identification of repetitive activation patterns using novel computational analysis of multielectrode recordings during atrial fibrillation and flutter in humans. *JACC Clin. Electrophysiol.* 3 207–216. 10.1016/j.jacep.2016.08.001 29759514

[B22] de Groot N. M. S. Houben R. P. M. Smeets J. L. Boersma E. Schotten U. Schalij M. J. (2010). Electropathological substrate of longstanding persistent atrial fibrillation in patients with structural heart disease: epicardial breakthrough. *Circulation* 122 1674–1682. 10.1161/circulationaha.109.910901 20937979

[B23] Dimitri H. Ng M. Brooks A. G. Kuklik P. Stiles M. K. Lau D. H. (2012). Atrial remodeling in obstructive sleep apnea: implications for atrial fibrillation. *Hear. Rhythm* 9 321–327. 10.1016/j.hrthm.2011.10.017 22016075

[B24] Dobrev D. Voigt N. Wehrens X. H. T. (2011). The ryanodine receptor channel as a molecular motif in atrial fibrillation: pathophysiological and therapeutic implications. *Cardiovasc. Res.* 89 734–743. 10.1093/cvr/cvq324 20943673 PMC3039246

[B25] Donal E. Lip G. Y. H. Galderisi M. Goette A. Shah D. Marwan M. (2016). EACVI/EHRA expert consensus document on the role of multi-modality imaging for the evaluation of patients with atrial fibrillation. *Eur. Heart J. Cardiovasc. Imaging* 17 355–383. 10.1093/ehjci/jev354 26864186

[B26] Everett T. H. IV Olgin J. E. (2007). Atrial fibrosis and the mechanisms of atrial fibrillation. *Hear. Rhythm* 4 22–24. 10.1016/j.hrthm.2006.12.040 PMC1850572 17336879

[B27] Franz M. R. Karasik P. L. Li C. Moubarak J. Chavez M. (1997). Electrical remodeling of the human atrium: similar effects in patients with chronic atrial fibrillation and atrial flutter. *J. Am. Coll. Cardiol.* 30 1785–1792. 10.1016/s0735-1097(97)00385-9 9385908

[B28] Fry C. H. Gray R. P. Dhillon P. S. Jabr R. I. Dupont E. Patel P. M. (2014). Architectural correlates of myocardial conduction: changes to the topography of cellular coupling, intracellular conductance, and action potential propagation with hypertrophy in Guinea-pig ventricular myocardium. *Circ. Arrhythmia Electrophysiol.* 7 1198–1204. 10.1161/circep.114.001471 25313260

[B29] Gerstenfeld E. P. Callans D. J. Dixit S. Zado E. Marchlinski F. E. (2003). Incidence and location of focal atrial fibrillation triggers in patients undergoing repeat pulmonary vein isolation: implications for ablation strategies. *J. Cardiovasc. Electrophysiol.* 14 685–690. 10.1046/j.1540-8167.2003.03013.x 12930245

[B30] Gibson D. N. Di Biase L. Mohanty P. Patel J. D. Bai R. Sanchez J. (2011). Stiff left atrial syndrome after catheter ablation for atrial fibrillation: clinical characterization, prevalence, and predictors. *Hear. Rhythm* 8 1364–1371. 10.1016/j.hrthm.2011.02.026 21354332

[B31] Gizurarson S. Dalvi R. Das M. Ha A. C. T. Suszko A. Chauhan V. S. (2016). Hierarchical schema for identifying focal electrical sources during human atrial fibrillation: implications for catheter-based atrial substrate ablation. *JACC Clin. Electrophysiol.* 2 656–666. 10.1016/j.jacep.2016.02.009 29759743

[B32] Goette A. Kalman J. M. Aguinaga L. Akar J. Cabrera J. A. Chen S. A. (2016). EHRA/HRS/APHRS/SOLAECE expert consensus on atrial cardiomyopathies: definition, characterization, and clinical implication. *Europace* 18 1455–1490. 10.1093/europace/euw161 27402624 PMC6392440

[B33] Gonzales M. J. Vincent K. P. Rappel W. J. Narayan S. M. McCulloch A. D. (2014). Structural contributions to fibrillatory rotors in a patient-derived computational model of the atria. *Europace* 16 iv3–iv10. 25362167 10.1093/europace/euu251 PMC4565557

[B34] Grace A. Verma A. Willems S. (2017). Dipole density mapping of atrial fibrillation. *Eur. Heart J.* 38 5–9. 10.1093/eurheartj/ehw585 28110304

[B35] Haissaguerre M. Hocini M. Denis A. Shah A. J. Komatsu Y. Yamashita S. (2014). Driver domains in persistent atrial fibrillation. *Circulation* 130 530–538. 25028391 10.1161/CIRCULATIONAHA.113.005421

[B36] Haïssaguerre M. Jaïs P. Shah D. C. Takahashi A. Hocini M. Quiniou G. (1998). Spontaneous initiation of atrial fibrillation by ectopic beats originating in the pulmonary veins. *N. Engl. J. Med.* 339 659–666. 10.1056/nejm199809033391003 9725923

[B37] Hansen B. J. Csepe T. A. Zhao J. Ignozzi A. J. Hummel J. D. Fedorov V. V. (2016). Maintenance of atrial fibrillation: are reentrant drivers with spatial stability the key? *Circ. Arrhythmia Electrophysiol.* 9 1–12. 10.1161/CIRCEP.116.004398 PMC5066578 27729340

[B38] Hansen B. J. Zhao J. Csepe T. A. Moore B. T. Li N. Jayne L. A. (2015). Atrial fibrillation driven by micro-anatomic intramural re-entry revealed by simultaneous sub-epicardial and sub-endocardial optical mapping in explanted human hearts. *Eur. Heart J.* 36 2390–2401. 10.1093/eurheartj/ehv233 26059724 PMC4568403

[B39] Hansen B. J. Zhao J. Fedorov V. V. (2017). Fibrosis and atrial fibrillation: computerized and optical mapping: a view into the human atria at submillimeter resolution. *JACC Clin. Electrophysiol.* 3 531–546. 10.1016/j.jacep.2017.05.002 29159313 PMC5693365

[B40] Hansen B. J. Zhao J. Li N. Zolotarev A. Zakharkin S. Wang Y. (2018). Human atrial fibrillation drivers resolved with integrated functional and structural imaging to benefit clinical mapping. *JACC Clin. Electrophysiol.* 4 1501–1515. 10.1016/j.jacep.2018.08.024 30573112 PMC6323649

[B41] Hayashi K. An Y. Nagashima M. Hiroshima K. Ohe M. Makihara Y. (2015). Importance of nonpulmonary vein foci in catheter ablation for paroxysmal atrial fibrillation. *Hear. Rhythm* 12 1918–1924. 10.1016/j.hrthm.2015.05.003 25962801

[B42] Heijman J. Voigt N. Nattel S. Dobrev D. (2014). Cellular and molecular electrophysiology of atrial fibrillation initiation, maintenance, and progression. *Circ. Res.* 114 1483–1499. 10.1161/circresaha.114.302226 24763466

[B43] Hojo R. Fukamizu S. Kitamura T. Aomyama Y. Nishizaki M. Kobayashi Y. (2017). Development of nonpulmonary vein foci increases risk of atrial fibrillation recurrence after pulmonary vein isolation. *JACC Clin. Electrophysiol.* 3 547–555. 10.1016/j.jacep.2016.12.008 29759426

[B44] Honarbakhsh S. Schilling R. J. Dhillon G. Ullah W. Keating E. Providencia R. (2018). A novel mapping system for panoramic mapping of the left atrium: application to detect and characterize localized sources maintaining atrial fibrillation. *JACC Clin. Electrophysiol.* 4 124–134. 29387810 10.1016/j.jacep.2017.09.177 PMC5777816

[B45] Iwasaki Y. Kato T. Xiong F. Shi Y.-F. Naud P. Maguy A. (2014). Atrial fibrillation promotion with long-term repetitive obstructive sleep apnea in a rat model. *J. Am. Coll. Cardiol.* 64 2013–2023. 10.1016/j.jacc.2014.05.077 25440097

[B46] Jadidi A. S. Lehrmann H. Keyl C. Sorrel J. Markstein V. Minners J. (2016). Ablation of persistent atrial fibrillation targeting low-voltage areas with selective activation characteristics. *Circ. Arrhythmia Electrophysiol.* 9 1–11. 10.1161/CIRCEP.115.002962 26966286

[B47] Jalife J. (2003). Rotors and spiral waves in atrial fibrillation. *J. Cardiovasc. Electrophysiol.* 14 776–780. 10.1046/j.1540-8167.2003.03136.x 12930260

[B48] January C. T. Wann L. S. Alpert J. S. Calkins H. Cigarroa J. E. Cleveland J. C. (2014). 2014 AHA/ACC/HRS guideline for the management of patients with atrial fibrillation: a report of the American college of Cardiology/American heart association task force on practice guidelines and the heart rhythm society. *J. Am. Coll. Cardiol.* 64 e1–e76. 24685669 10.1016/j.jacc.2014.03.022

[B49] January C. T. Wann L. S. Calkins H. Chen L. Y. Cigarroa J. E. Cleveland J. C. (2019). 2019 AHA/ACC/HRS focused update of the 2014 AHA/ACC/HRS guideline for the management of patients with atrial fibrillation: a report of the american college of cardiology/American heart association task force on clinical practice guidelines and the heart R. *Hear. Rhythm* 16 e66–e93. 10.1016/j.hrthm.2019.01.024 30703530

[B50] Josephson M. E. Anter E. (2015). Substrate mapping for ventricular tachycardia: assumptions and misconceptions. *JACC Clin. Electrophysiol.* 1 341–352. 29759461 10.1016/j.jacep.2015.09.001

[B51] Kapa S. Desjardins B. Callans D. J. Marchlinski F. E. Dixit S. (2014). Contact electroanatomic mapping derived voltage criteria for characterizing left atrial scar in patients undergoing ablation for atrial fibrillation. *J. Cardiovasc. Electrophysiol.* 25 1044–1052. 10.1111/jce.12452 24832482

[B52] Karim R. Housden R. J. Balasubramaniam M. Chen Z. Perry D. Uddin A. (2013). Evaluation of current algorithms for segmentation of scar tissue from late Gadolinium enhancement cardiovascular magnetic resonance of the left atrium: an open-access grand challenge. *J. Cardiovasc. Magn. Reson.* 15 1–17. 24359544 10.1186/1532-429X-15-105 PMC3878126

[B53] Kircher S. Arya A. Altmann D. Rolf S. Bollmann A. Sommer P. (2018). Individually tailored vs. standardized substrate modification during radiofrequency catheter ablation for atrial fibrillation: a randomized study. *Europace* 20 1766–1775. 10.1093/europace/eux310 29177475

[B54] Knecht S. Sohal M. Deisenhofer I. Albenque J. P. Arentz T. Neumann T. (2017). Multicentre evaluation of non-invasive biatrial mapping for persistent atrial fibrillation ablation: the AFACART study. *Europace* 19 1302–1309. 10.1093/europace/euw168 28204452

[B55] Kottkamp H. (2013). Human atrial fibrillation substrate: towards a specific fibrotic atrial cardiomyopathy. *Eur. Heart J.* 34 2731–2738. 10.1093/eurheartj/eht194 23761394

[B56] Kottkamp H. Berg J. Bender R. Rieger A. Schreiber D. (2016). Box isolation of fibrotic areas (BIFA): a patient-tailored substrate modification approach for ablation of atrial fibrillation. *J. Cardiovasc. Electrophysiol.* 27 22–30. 10.1111/jce.12870 26511713

[B57] Kowalewski C. A. B. Shenasa F. Rodrigo M. Clopton P. Meckler G. Alhusseini M. I. (2018). Interaction of localized drivers and disorganized activation in persistent atrial fibrillation: reconciling putative mechanisms using multiple mapping techniques. *Circ. Arrhythmia Electrophysiol.* 11 1–12. 10.1161/CIRCEP.117.005846 PMC6475887 29884620

[B58] Krummen D. E. Bayer J. D. Ho J. Ho G. Smetak M. R. Clopton P. (2012). Mechanisms of human atrial fibrillation initiation clinical and computational studies of repolarization restitution and activation latency. *Circ. Arrhythmia Electrophysiol.* 5 1149–1159. 10.1161/circep.111.969022 23027797 PMC3833353

[B59] Krummen D. E. Hebsur S. Salcedo J. Narayan S. M. Lalani G. G. Schricker A. A. (2015). Mechanisms underlying AF: triggers, rotors, other? *Curr. Treat. Options Cardiovasc. Med.* 17:371. 10.1007/s11936-015-0371-4 PMC4800994 25778423

[B60] Lalani G. G. Schricker A. Gibson M. Rostamian A. Krummen D. E. Narayan S. M. (2012). Atrial conduction slows immediately before the onset of human atrial fibrillation: a bi-atrial contact mapping study of transitions to atrial fibrillation. *J. Am. Coll. Cardiol.* 59 595–606. 10.1016/j.jacc.2011.10.879 22300695 PMC3390156

[B61] Lalani G. G. Trikha R. Krummen D. E. Narayan S. M. (2014). Rotors and focal sources for human atrial fibrillation–Mechanistic paradigm with direct clinical relevance. *Circ. J.* 78 2357–2366. 10.1253/circj.cj-14-0478 25213002

[B62] Lau D. H. Nattel S. Kalman J. M. Sanders P. (2017). Modifiable risk factors and atrial fibrillation. *Circulation* 136 583–596. 10.1161/circulationaha.116.023163 28784826

[B63] Leef G. Shenasa F. Bhatia N. K. Rogers A. J. Sauer W. Miller J. M. (2019). Wavefront field mapping reveals a physiologic network between drivers where ablation terminates atrial fibrillation. *Circ. Arrhythmia Electrophysiol.* 12 1–10. 10.1161/CIRCEP.118.006835 PMC6666420 31352796

[B64] Li D. Fareh S. Leung T. K. Nattel S. (1999). Promotion of atrial fibrillation by heart failure in dogs: atrial remodeling of a different sort. *Circulation* 100 87–95. 10.1161/01.cir.100.1.87 10393686

[B65] Li D. Zhang L. Kneller J. Nattel S. (2001). Potential ionic mechanism for repolarization differences between canine right and left atrium. *Circ. Res.* 88 1168–1175. 10.1161/hh1101.091266 11397783

[B66] Lin W. S. Tai C. T. Hsieh M. H. Tsai C. F. Lin Y. K. Tsao H. M. (2003). Catheter ablation of paroxysmal atrial fibrillation initiated by non-pulmonary vein ectopy. *Circulation* 107 3176–3183. 10.1161/01.cir.0000074206.52056.2d 12821558

[B67] Ling Z. McManigle J. Zipunnikov V. Pashakhanloo F. Khurram I. M. Zimmerman S. L. (2015). The association of left atrial low-voltage regions on electroanatomic mapping with low attenuation regions on cardiac computed tomography perfusion imaging in patients with atrial fibrillation. *Hear. Rhythm* 12 857–864. 10.1016/j.hrthm.2015.01.015 25595922 PMC4410061

[B68] Mahajan R. Lau D. H. Brooks A. G. Shipp N. J. Manavis J. Wood J. P. M. (2015). Electrophysiological, electroanatomical, and structural remodeling of the atria as consequences of sustained obesity. *J. Am. Coll. Cardiol.* 66 1–11. 10.1016/j.jacc.2015.04.058 26139051

[B69] Mahnkopf C. Badger T. J. Burgon N. S. Daccarett M. Haslam T. S. Badger C. T. (2010). Evaluation of the left atrial substrate in patients with lone atrial fibrillation using delayed-enhanced MRI: implications for disease progression and response to catheter ablation. *Hear. Rhythm* 7 1475–1481. 10.1016/j.hrthm.2010.06.030 20601148 PMC3106345

[B70] Malcolme-Lawes L. C. Juli C. Karim R. Bai W. Quest R. Lim P. B. (2013). Automated analysis of atrial late gadolinium enhancement imaging that correlates with endocardial voltage and clinical outcomes: A 2-center study. *Hear. Rhythm* 10 1184–1191. 10.1016/j.hrthm.2013.04.030 23685170 PMC3734347

[B71] Marrouche N. F. Wilber D. Hindricks G. Jais P. Akoum N. Marchlinski F. (2014). Association of atrial tissue fibrosis identified by delayed enhancement MRI and atrial fibrillation catheter ablation: the DECAAF study. *JAMA J. Am. Med. Assoc.* 311 498–506. 10.1001/jama.2014.3 24496537

[B72] McDowell K. S. Vadakkumpadan F. Blake R. Blauer J. Plank G. Macleod R. S. (2013). Mechanistic inquiry into the role of tissue remodeling in fibrotic lesions in human atrial fibrillation. *Biophys. J.* 104 2764–2773. 10.1016/j.bpj.2013.05.025 23790385 PMC3686346

[B73] McDowell K. S. Zahid S. Vadakkumpadan F. Blauer J. MacLeod R. S. Trayanova N. A. (2015). Virtual electrophysiological study of atrial fibrillation in fibrotic remodeling. *PLoS One* 10 1–16. 10.1371/journal.pone.0117110 PMC4333565 25692857

[B74] McGann C. Akoum N. Patel A. Kholmovski E. Revelo P. Damal K. (2014). Atrial fibrillation ablation outcome is predicted by left atrial remodeling on MRI. *Circ. Arrhythmia Electrophysiol.* 7 23–30. 10.1161/CIRCEP.113.000689 PMC4086672 24363354

[B75] Miller J. M. Kalra V. Das M. K. Jain R. Garlie J. B. Brewster J. A. (2017). Clinical benefit of ablating localized sources for human atrial fibrillation: the indiana university FIRM registry. *J. Am. Coll. Cardiol.* 69 1247–1256. 10.1016/j.jacc.2016.11.079 28279291

[B76] Miller J. M. Kowal R. C. Swarup V. Daubert J. P. Daoud E. G. Day J. D. (2014). Initial independent outcomes from focal impulse and rotor modulation ablation for atrial fibrillation: multicenter FIRM registry. *J. Cardiovasc. Electrophysiol.* 25 921–929. 10.1111/jce.12474 24948520 PMC4282180

[B77] Morgan R. Colman M. A. Chubb H. Seemann G. Aslanidi O. V. (2016). Slow conduction in the border zones of patchy fibrosis stabilizes the drivers for atrial fibrillation: Insights from multi-scale human atrial modeling. *Front. Physiol.* 7 1–15. 27826248 10.3389/fphys.2016.00474 PMC5079097

[B78] Munger T. M. Dong Y.-X. Masaki M. Oh J. K. Mankad S. V. Borlaug B. A. (2012). Electrophysiological and hemodynamic characteristics associated with obesity in patients with atrial fibrillation. *J. Am. Coll. Cardiol.* 60 851–860. 22726633 10.1016/j.jacc.2012.03.042

[B79] Narayan S. M. Baykaner T. Clopton P. Schricker A. Lalani G. G. Krummen D. E. (2014). Ablation of rotor and focal sources reduces late recurrence of atrial fibrillation compared with trigger ablation alone: extended follow-up of the CONFIRM trial (conventional ablation for atrial fibrillation with or without focal impulse and rotor modulat. *J. Am. Coll. Cardiol.* 63 1761–1768. 10.1016/j.jacc.2014.02.543 24632280 PMC4008643

[B80] Narayan S. M. Kazi D. Krummen D. E. Rappel W. J. (2008). Repolarization and activation restitution near human pulmonary veins and atrial fibrillation initiation: a mechanism for the initiation of atrial fibrillation by premature beats. *J. Am. Coll. Cardiol.* 52 1222–1230. 10.1016/j.jacc.2008.07.012 18926325 PMC2604131

[B81] Narayan S. M. Krummen D. E. Enyeart M. W. Rappel W. J. (2012a). Computational mapping identifies localized mechanisms for ablation of atrial fibrillation. *PLoS One* 7:e46034. 10.1371/journal.pone.0046034 23049929 PMC3458823

[B82] Narayan S. M. Krummen D. E. Rappel W. J. (2012b). Clinical mapping approach to diagnose electrical rotors and focal impulse sources for human atrial fibrillation. *J. Cardiovasc. Electrophysiol.* 23 447–454. 10.1111/j.1540-8167.2012.02332.x 22537106 PMC3418865

[B83] Narayan S. M. Krummen D. E. Shivkumar K. Clopton P. Rappel W. J. Miller J. M. (2012c). Treatment of atrial fibrillation by the ablation of localized sources: CONFIRM (Conventional Ablation for Atrial Fibrillation with or Without Focal Impulse and Rotor Modulation) trial. *J. Am. Coll. Cardiol.* 60 628–636. 22818076 10.1016/j.jacc.2012.05.022 PMC3416917

[B84] Narayan S. M. Patel J. Mulpuru S. Krummen D. E. (2012d). Focal impulse and rotor modulation ablation of sustaining rotors abruptly terminates persistent atrial fibrillation to sinus rhythm with elimination on follow-up: a video case study. *Hear. Rhythm* 9 1436–1439. 10.1016/j.hrthm.2012.03.055 22465458 PMC3432749

[B85] Narayan S. M. Shivkumar K. Krummen D. E. Miller J. M. Rappel W. J. (2013). Panoramic electrophysiological mapping but not electrogram morphology identifies stable sources for human atrial fibrillation: stable atrial fibrillation rotors and focal sources relate poorly to fractionated electrograms. *Circ. Arrhythm. Electrophysiol.* 6 58–67. 10.1161/circep.111.977264 23392583 PMC3746540

[B86] Nattel S. Burstein B. Dobrev D. (2008). Atrial remodeling and atrial fibrillation: mechanisms and implications. *Circ. Arrhythmia Electrophysiol.* 1 62–73. 10.1161/circep.107.754564 19808395

[B87] Nattel S. Xiong F. Aguilar M. (2017). Demystifying rotors and their place in clinical translation of atrial fibrillation mechanisms. *Nat. Rev. Cardiol.* 14 509–520. 10.1038/nrcardio.2017.37 28383023

[B88] Numata A. Miyauchi Y. Ono N. Fishbein M. C. Mandel W. J. Lin S. (2012). Spontaneous atrial fibrillation initiated by tyramine in canine atria with increased sympathetic nerve sprouting. *J. Cardiovasc. Electrophysiol.* 23 415–422. 10.1111/j.1540-8167.2011.02197.x 22034958 PMC4364657

[B89] Oakes R. S. Badger T. J. Kholmovski E. G. Akoum N. Burgon N. S. Fish E. N. (2009). Detection and quantification of left atrial structural remodeling with delayed-enhancement magnetic resonance imaging in patients with atrial fibrillation. *Circulation* 119 1758–1767. 10.1161/circulationaha.108.811877 19307477 PMC2725019

[B90] Packer D. L. Mark D. B. Robb R. A. Monahan K. H. Bahnson T. D. Poole J. E. (2019). Effect of catheter ablation vs antiarrhythmic drug therapy on mortality, stroke, bleeding, and cardiac arrest among patients with atrial fibrillation: the CABANA randomized clinical trial. *JAMA J. Am. Med. Assoc.* 321 1261–1274. 10.1001/jama.2019.0693 PMC6450284 30874766

[B91] Pashakhanloo F. Herzka D. A. Ashikaga H. Mori S. Gai N. Bluemke D. A. (2016). Myofiber architecture of the human atria as revealed by submillimeter diffusion tensor imaging. *Circ. Arrhythmia Electrophysiol.* 9:e004133. 10.1161/CIRCEP.116.004133 PMC7035884 27071829

[B92] Pathak R. K. Middeldorp M. E. Lau D. H. Mehta A. B. Mahajan R. Twomey D. (2014). Aggressive risk factor reduction study for atrial fibrillation and implications for the outcome of ablation: the ARREST-AF cohort study. *J. Am. Coll. Cardiol.* 64 2222–2231. 10.1016/j.jacc.2014.09.028 25456757

[B93] Pathak R. K. Middeldorp M. E. Meredith M. Mehta A. B. Mahajan R. Wong C. X. (2015). Long-term effect of goal-directed weight management in an atrial fibrillation cohort: a long-term follow-up study (LEGACY). *J. Am. Coll. Cardiol.* 65 2159–2169. 25792361 10.1016/j.jacc.2015.03.002

[B94] Pérez F. J. Schubert C. M. Parvez B. Pathak V. Ellenbogen K. A. Wood M. A. (2009). Long-term outcomes after catheter ablation of cavo-tricuspid isthmus dependent atrial flutter: a meta-analysis. *Circ. Arrhythmia Electrophysiol.* 2 393–401. 10.1161/circep.109.871665 19808495

[B95] Qiao Y. Shi R. Hou B. Wu L. Zheng L. Ding L. (2015). Impact of alcohol consumption on substrate remodeling and ablation outcome of paroxysmal atrial fibrillation. *J. Am. Heart Assoc.* 4:e002349. 10.1161/JAHA.115.002349 PMC4845226 26553213

[B96] Quintanilla J. G. Pérez-Villacastín J. Pérez-Castellano N. Pandit S. V. Berenfeld O. Jalife J. (2016). Mechanistic approaches to detect, target, and ablate the drivers of atrial fibrillation. *Circ. Arrhythmia Electrophysiol.* 9 1–11. 10.1161/CIRCEP.115.002481 PMC5547888 26729850

[B97] Ramanathan C. Ghanem R. N. Jia P. Ryu K. Rudy Y. (2004). Noninvasive electrocardiographic imaging for cardiac electrophysiology and arrhythmia. *Nat. Med.* 10 422–428. 10.1038/nm1011 15034569 PMC1950745

[B98] Rolf S. Kircher S. Arya A. Eitel C. Sommer P. Sergio R. (2014). Tailored atrial substrate modification based on low-voltage areas in catheter ablation of atrial fibrillation. *Circ. Arrhythmia Electrophysiol.* 7 825–833. 10.1161/circep.113.001251 25151631

[B99] Saeed M. Van T. A. Krug R. Hetts S. W. Wilson M. W. (2015). Cardiac MR imaging: current status and future direction. *Cardiovasc. Diagn. Ther.* 5 290–310. 26331113 10.3978/j.issn.2223-3652.2015.06.07 PMC4536478

[B100] Santangeli P. Zado E. S. Hutchinson M. D. Riley M. P. Lin D. Frankel D. S. (2016). Prevalence and distribution of focal triggers in persistent and long-standing persistent atrial fibrillation. *Hear. Rhythm* 13 374–382. 10.1016/j.hrthm.2015.10.023 26477712

[B101] Sauer W. H. Alonso C. Zado E. Cooper J. M. Lin D. Dixit S. (2006). Atrioventricular nodal reentrant tachycardia in patients referred for atrial fibrillation ablation: response to ablation that incorporates slow-pathway modification. *Circulation* 114 191–195. 10.1161/circulationaha.106.621896 16831982

[B102] Schade A. Nentwich K. Costello-Boerrigter L. C. Halbfass P. Mueller P. Roos M. (2016). Spatial relationship of focal impulses, rotors and low voltage zones in patients with persistent atrial fibrillation. *J. Cardiovasc. Electrophysiol.* 27 507–514. 10.1111/jce.12913 26732468

[B103] Schricker A. A. Lalani G. G. Krummen D. E. Rappel W. J. Narayan S. M. (2014). Human atrial fibrillation initiates via organized rather than disorganized mechanisms. *Circ. Arrhythm Electrophysiol.* 7 816–824. 10.1161/circep.113.001289 25217042 PMC4206587

[B104] Seitz J. Bars C. Théodore G. Beurtheret S. Lellouche N. Bremondy M. (2017). AF ablation guided by spatiotemporal electrogram dispersion without pulmonary vein isolation: a wholly patient-tailored approach. *J. Am. Coll. Cardiol.* 69 303–321. 10.1016/j.jacc.2016.10.065 28104073 PMC5568427

[B105] Shen M. J. Arora R. Jalife J. (2019). Atrial myopathy. *JACC Basic Transl. Sci.* 4 640–654. 31768479 10.1016/j.jacbts.2019.05.005 PMC6872845

[B106] Shi R. Parikh P. Chen Z. Angel N. Norman M. Hussain W. (2020). Validation of dipole density mapping during atrial fibrillation and sinus rhythm in human left atrium. *JACC Clin. Electrophysiol.* 6 171–181. 10.1016/j.jacep.2019.09.012 32081219

[B107] Shin S. Y. Jo W.-M. Min T. J. Kim B.-K. Song D. H. Hyeon S. H. (2015). Gap junction remodelling by chronic pressure overload is related to the increased susceptibility to atrial fibrillation in rat heart. *Ep. Eur.* 17 655–663. 10.1093/europace/euu294 25398404

[B108] Shivkumar K. Ellenbogen K. A. Hummel J. D. Miller J. M. Steinberg J. S. (2012). Acute termination of human atrial fibrillation by identification and catheter ablation of localized rotors and sources: first multicenter experience of focal impulse and rotor modulation (FIRM) ablation. *J. Cardiovasc. Electrophysiol.* 23 1277–1285. 10.1111/jce.12000 23130890 PMC3524347

[B109] Sohns C. Marrouche N. F. (2020). Atrial fibrillation and cardiac fibrosis. *Eur. Heart J.* 41 1123–1131. 10.1093/eurheartj/ehz786 31713590

[B110] Spragg D. D. Khurram I. Zimmerman S. L. Yarmohammadi H. Barcelon B. Needleman M. (2012). Initial experience with magnetic resonance imaging of atrial scar and co-registration with electroanatomic voltage mapping during atrial fibrillation: success and limitations. *Hear. Rhythm* 9 2003–2009. 10.1016/j.hrthm.2012.08.039 23000671

[B111] Stiles M. K. John B. Wong C. X. Kuklik P. Brooks A. G. Lau D. H. (2009). Paroxysmal lone atrial fibrillation is associated with an abnormal atrial substrate. Characterizing the ‘second factor’. *J. Am. Coll. Cardiol.* 53 1182–1191. 19341858 10.1016/j.jacc.2008.11.054

[B112] Takigawa M. Takahashi A. Kuwahara T. Okubo K. Takahashi Y. Nakashima E. (2015). Impact of non-pulmonary vein foci on the outcome of the second session of catheter ablation for paroxysmal atrial fibrillation. *J. Cardiovasc. Electrophysiol.* 26 739–746. 10.1111/jce.12681 25845757

[B113] Van Campenhout M. J. H. Yaksh A. Kik C. De Jaegere P. P. Ho S. Y. Allessie M. A. (2013). Bachmann’s bundle a key player in the development of atrial fibrillation? *Circ. Arrhythmia Electrophysiol.* 6 1041–1046. 10.1161/circep.113.000758 24129206

[B114] Verma A. Jiang C. Y. Betts T. R. Chen J. Deisenhofer I. Mantovan R. (2015). Approaches to catheter ablation for persistent atrial fibrillation. *N. Engl. J. Med.* 372 1812–1822. 25946280 10.1056/NEJMoa1408288

[B115] Verma A. Wazni O. M. Marrouche N. F. Martin D. O. Kilicaslan F. Minor S. (2005). Pre-existent left atrial scarring in patients undergoing pulmonary vein antrum isolation: an independent predictor of procedural failure. *J. Am. Coll. Cardiol.* 45 285–292. 10.1016/j.jacc.2004.10.035 15653029

[B116] Voskoboinik A. Prabhu S. Ling L. Kalman J. M. Kistler P. M. (2016). Alcohol and atrial fibrillation: a sobering review. *J. Am. Coll. Cardiol.* 68 2567–2576. 27931615 10.1016/j.jacc.2016.08.074

[B117] Wijffels M. C. E. F. Kirchhof C. J. H. J. Dorland R. Allessie M. A. (1995). Atrial fibrillation begets atrial fibrillation: a study in awake chronically instrumented goats. *Circulation* 92 1954–1968. 10.1161/01.cir.92.7.1954 7671380

[B118] Willems S. Verma A. Betts T. R. Murray S. Neuzil P. Ince H. (2019). Targeting nonpulmonary vein sources in persistent atrial fibrillation identified by noncontact charge density mapping: UNCOVER AF trial. *Circ. Arrhythmia Electrophysiol.* 12 1–12. 10.1161/CIRCEP.119.007233 31242746

[B119] Yagishita A. Gimbel J. R. De Oliveira S. Manyam H. Sparano D. Cakulev I. (2017). Long-term outcome of left atrial voltage-guided substrate ablation during atrial fibrillation: a novel adjunctive ablation strategy. *J. Cardiovasc. Electrophysiol.* 28 147–155. 10.1111/jce.13122 27862561

[B120] Yue L. Xie J. Nattel S. (2011). Molecular determinants of cardiac fibroblast electrical function and therapeutic implications for atrial fibrillation. *Cardiovasc. Res.* 89 744–753. 10.1093/cvr/cvq329 20962103 PMC3039247

[B121] Zahid S. Cochet H. Boyle P. M. Schwarz E. L. Whyte K. N. Vigmond E. J. (2016). Patient-derived models link re-entrant driver localization in atrial fibrillation to fibrosis spatial pattern. *Cardiovasc. Res.* 110 443–454. 10.1093/cvr/cvw073 27056895 PMC4872878

[B122] Zaman J. A. B. Narayan S. M. (2015). When is structure, function? Revisiting an old concept in atrial fibrillation. *J. Cardiovasc. Electrophysiol.* 26 1361–1363. 10.1111/jce.12836 26359793 PMC4764076

[B123] Zaman J. A. B. Sauer W. H. Alhusseini M. I. Baykaner T. Borne R. T. Kowalewski C. A. B. (2018). Identification and characterization of sites where persistent atrial fibrillation is terminated by localized ablation. *Circ. Arrhythmia Electrophysiol.* 11 1–12. 10.1161/CIRCEP.117.005258 PMC5769709 29330332

[B124] Zellerhoff S. Pistulli R. Mönnig G. Hinterseer M. Beckmann B. Koebe J. (2009). Atrial arrhythmias in long−QT syndrome under daily life conditions: a nested case control study. *J. Cardiovasc. Electrophysiol.* 20 401–407. 10.1111/j.1540-8167.2008.01339.x 19017345

[B125] Zhao J. Hansen B. J. Wang Y. Csepe T. A. Sul L. V. Tang A. (2017). Three-dimensional integrated functional, structural, and computational mapping to define the structural ‘fingerprints’ of heart-specific atrial fibrillation drivers in human heart ex vivo. *J. Am. Heart Assoc.* 6:e005922. 10.1161/JAHA.117.005922 PMC5586436 28862969

